# Lymphocytes and immunoglobulins in peripheral blood and lymphatic fluid of neonates with chylothorax

**DOI:** 10.3389/fimmu.2025.1666366

**Published:** 2025-11-03

**Authors:** Domenico Umberto De Rose, Francesca Landolfo, Flaminia Pugnaloni, Fatima Zahra Gassabi, Ludovica Martini, Alessandra Santisi, Claudia Columbo, Paola Giliberti, Fabia Gazzotti, Ottavia Porzio, Claudia Capponi, Carlo Federico Perno, Maria Paola Ronchetti, Andrea Conforti, Guglielmo Salvatori, Irma Capolupo, Andrea Dotta

**Affiliations:** ^1^ Neonatal Intensive Care Unit – “Bambino Gesù” Children’s Hospital IRCCS, Rome, Italy; ^2^ Faculty of Medicine and Surgery, “Tor Vergata” University of Rome, Rome, Italy; ^3^ Clinical Chemistry Laboratory Unit – “Bambino Gesù” Children’s Hospital IRCCS, Rome, Italy; ^4^ Department of Experimental Medicine, University of Rome “Tor Vergata”, Rome, Italy; ^5^ Microbiology and Diagnostic Immunology Unit – “Bambino Gesù” Children’s Hospital IRCCS, Rome, Italy; ^6^ Neonatal and Pediatric Surgery Unit – “Bambino Gesù” Children’s Hospital IRCCS, Rome, Italy

**Keywords:** late-onset sepsis, chyle, T-cells, IgG, infection, fungi, bacteria

## Abstract

**Background:**

Neonatal chylothorax is associated with high morbidity and mortality, partly due to increased infection risk from lymphocyte depletion and hypogammaglobulinemia. However, data specific to chylothorax cohorts are limited. This study aimed to investigate lymphocyte subsets and immunoglobulin levels in neonates with congenital and acquired chylothorax.

**Methods:**

We retrospectively enrolled 18 neonates with chylothorax admitted to our NICU between January 2023 and January 2025. Inclusion criteria were term or preterm infants with congenital or acquired chylothorax and paired peripheral blood and chyle samples collected within 48 hours of effusion onset. Lymphocyte subsets and immunoglobulin levels were compared between blood and chyle, and between congenital and acquired chylothorax.

**Results:**

Chyle samples showed significantly higher leukocyte, lymphocyte (percentage and absolute), and T-cell counts compared to blood. Conversely, B lymphocyte percentages and Natural killer (NK) cell counts were significantly lower in chyle, as were IgG and IgM levels. Three patients (16.7%) had absolute lymphopenia, particularly within the T-cell and NK-cell subsets, and five (27.8%) had hypogammaglobulinemia. Fifteen infants (83.3%) developed late-onset sepsis, primarily bacterial, with some fungal cases. Absolute T-cell subset numbers in chyle were higher in acquired versus congenital chylothorax, while percentages and immunoglobulin levels were largely similar.

**Conclusion:**

Our findings confirm a specific pattern of immune dysregulation in neonates with chylothorax, with distinct lymphocyte and immunoglobulin profiles in chyle. In particular, the T-cell subsets were enriched in the chyle. A multicenter, prospective randomized trial is warranted to assess the utility of immunoglobulin therapy in infection prevention in this population.

## Introduction

Chylothorax is defined as an effusion of lymph in the pleural cavity, with a triglyceride concentration >1.1 mmol/L (about 110 mg/dL), a total cell count > 1,000 cells/µL, and a lymphocyte predominance (>80 %) (1). It’s a rather uncommon condition in children: it can have serious clinical consequences, especially in neonates. It can be congenital or acquired, and it usually happens as a consequence of surgery for congenital heart disease or thoracic anomalies (2, 3).

The lymphatic system plays a critical role in maintaining fluid homeostasis, absorbing dietary fats, and supporting immune function. When this system is disrupted, as in the case of chylothorax, the resulting accumulation of chyle in the pleural space may compromise respiratory function and immune defense, with respiratory failure and increased risk of infections. Indeed, chylothorax is associated with significant morbidity and increased mortality risk (3). In particular, secondary immunodeficiency resulting from lymphocyte and immunoglobulin loss in chylous fluid may have important implications for infection risk, clinical management, and long-term outcomes (4, 5). This condition predisposes infants to an increased risk of bloodstream infections (6).

The main controversy concerns the efficacy and indication of routine intravenous immunoglobulin (IVIG) administration in neonates with chylothorax. Existing studies are few, often with small sample sizes and inconsistent results, leaving open the debate about the true therapeutic value and the optimal timing for administration (7). In particular, studies evaluating infants with chylothorax are few and have described small sample sizes (5, 8, 9).

The treatment strategy for neonatal chylothorax generally involves enteral fasting and supportive care; it also includes drainage and different therapeutic strategies to reduce chyle flow (such as octreotide) (6).

Studying the specific immunological alterations in patients with chylothorax, particularly the loss of immunoglobulins and lymphocytes, is crucial to understanding the real infection risk and vulnerabilities of these neonates. This knowledge can guide more targeted therapeutic decisions, such as appropriate and timely use of immunoglobulins, especially in infants with specific losses of immune cells required for humoral immunity response, potentially improving clinical management, reducing infectious complications, and enhancing long-term outcomes.

The aim of the present study was to determine whether deficiencies in lymphocyte subsets and immunoglobulin levels occur in patients with congenital and acquired chylothorax. To achieve this, we examined peripheral blood and chyle samples in a cohort of neonates with chylothorax. These data can help identify significant differences between the two groups and better define the immune system associated with the disease. This could clarify if and when immunoglobulin therapy is justified and support the development of more precise guidelines for immunological management in these patients.

## Materials and methods

### Study design and population

In this retrospective study, we consecutively enrolled all neonates and infants with chylothorax from January 2023 to January 2025, in whom lymphatic fluid was analyzed.

We consecutively enrolled all term and preterm neonates admitted to our Neonatal Intensive Care Unit with the following characteristics: 1) congenital or acquired chylothorax; 2) the availability of the measurement of lymphocyte subsets and immunoglobulin levels in a paired peripheral blood sample and a chyle sample per patient (simultaneously taken within 48 hours of pleural effusion onset). Patients who were conservatively managed (i.e., without drainage) were not included in this analysis, as our data collection was specifically focused on samples obtained from pleural drainage.

All blood and chyle samples used for the study’s immunological and biochemical analyses were collected before the initiation of any albumin infusion or other therapeutic interventions.

For the diagnosis of neonatal sepsis, we used clinical, biological, and microbiological parameters: postnatal signs of sepsis and a positive central and/or peripheral blood culture, taking into account both the recommendations of the US Centers for Disease Control for infants under 1 year of age (10), and the modified definitions on the newborn, which we have applied in all studies on neonatal sepsis, carried out by our group over the years (11, 12). Late-onset sepsis is a bloodstream infection in neonates occurring after 72 hours of life, acquired after being hospitalized for at least 48 hours.

Chylothorax was diagnosed based on the following criteria: ≥ 1,000 cells/µL in pleural fluid, with a lymphocyte fraction > 80%, and a triglyceride concentration > 110 mg/dL (1).

For each enrolled neonate, we collected clinical and anamnestic information stored in a dedicated electronic database.

### Aims of the study

The primary aim was to evaluate lymphocyte subsets and immunoglobulin levels in blood and chyle samples in infants with chylothorax.

The secondary aim was to evaluate differences in lymphocyte subsets and immunoglobulin levels in chyle samples in infants with congenital or acquired chylothorax.

Additionally, we aimed to evaluate how many late-onset sepsis episodes occurred in these infants and which pathogens were involved.

### Lymphocyte subsets and immunoglobulin measurement

Lymphocyte subset enumeration was performed by adding 20 µL of BD Multitest™ 6-Color TBNK reagent to BD Trucount™ tubes, followed by the addition of 50 µL of well-mixed, anticoagulated whole blood. Tubes were capped, gently vortexed, and incubated for 15–30 minutes at room temperature (20–25°C) in the dark. Subsequently, 450 µL of 1X BD FACS™ Lysing Solution was added, and samples were vortexed and incubated for an additional 15–30 minutes under the same conditions. Acquisition was carried out on the flow cytometer BD FACSLyric™ (BD Biosciences, USA), obtaining the values of T lymphocytes (CD3^+^), B lymphocytes (CD19^+^), Natural killer (NK) lymphocytes (CD3^–^CD16^+^ and/or CD56^+^), Helper/inducer T lymphocytes (CD3^+^CD4^+^), Suppressor/cytotoxic T lymphocytes (CD3^+^CD8^+^). Percentages of T, B, and NK lymphocytes were calculated relative to the total lymphocyte population. CD4^+^ and CD8^+^ T cell subsets were expressed as percentages of CD3^+^ T cells. Absolute counts were determined using the BD Trucount™ bead-based internal standard. Lymphocyte values were compared with published normal reference data for lymphocyte subsets in peripheral blood samples during neonatal age (13). For our analysis of lymphocyte subsets (e.g., T-cells, B-cells, NK-cells), absolute lymphopenia was defined as an absolute count of the specific lymphocyte subset below the 5th percentile for the corresponding neonatal age group, based on published reference data. Similarly, relative lymphopenia was defined as a percentage of that subset within the total lymphocyte population (or within CD3^+^ T cells for CD4^+^ and CD8^+^ subsets) below the respective reference percentile.

Immunoglobulin IgA, IgG, and IgM levels were analyzed by the immunoturbidimetric method (Cobas 8000, Roche Diagnostics, Mannheim, Germany). Measurement intervals for the Cobas^®^ analyzer were: IgA 0.04 – 8.00 g/L; IgG 0.007-50.0 g/L; IgM 0.05 – 6.50 g/L. Immunoglobulin levels were compared with published normal reference data for immunoglobulins in peripheral blood samples during the neonatal age (14).

### Statistical analysis

Data are presented as numbers and percentages for categorical variables. Continuous variables are expressed as the mean and standard deviation (SD), or as the median and interquartile range (IQR), according to the normality of data as assessed by the D’Agostino-Pearson test.

Differences in lymphocyte subsets and immunoglobulin levels in paired blood and chyle samples were assessed using the Wilcoxon Signed-Rank Test for Correlated Samples, as appropriate, because the comparisons were made within the same individuals.

Differences in lymphocyte subsets and immunoglobulin levels in chyle samples between infants with congenital and acquired chylothorax were assessed using the Mann-Whitney test, as appropriate for comparing two independent groups.

Finally, using the t-test for unpaired samples, we compared late-onset sepsis episodes that occurred in infants with hypogammaglobulinemia and infants without, and late-onset sepsis episodes that occurred in infants who received IVIG versus their peers who did not.

A *p*-value < 0.05 was considered significant. The statistical analysis was performed using IBM SPSS Statistics 25 (New York, USA).

## Results

### Patient characteristics and clinical course

We enrolled 18 patients with congenital or acquired chylothorax during the study period. Baseline characteristics of included patients were reported in **Table 1**.

**Table 1:** Baseline characteristics of included patients.

Variables	Patients (n=18)
Males, n (%)	11 (61.1%)
Gestational age, weeks	35 (33 – 38)
Birthweight, grams	2550 ± 772
Cause of hospitalization	Congenital causes (n= 4): 3 Congenital chylothorax and fetal hydrops 1 Congenital chylothorax with fetal ascites and intestinal lymphangioma Acquired causes (n= 14): 9 Congenital diaphragmatic hernia 2 Esophageal atresia 1 Transposition of great arteries 2 Preterm with intestinal perforation
Age at diagnosis of chylothorax, days	13 (7 – 18)
Weight at diagnosis of chylothorax, grams	2809 ± 684
Duration of mechanical ventilation, days	16 (8 – 23)
Need for high-frequency oscillatory ventilation, n (%)	11 (61.1%)
Duration of non-invasive ventilation, days	12 (6 – 29)
Need for oxygen, days	18 (3 – 32)
Use of octreotide, n (%)	13 (72.2%)
Age at surgery for surgical infants, hours	73 (48 – 114)
Weight at surgery for surgical infants, grams	2768 ± 959
Weight at full enteral feeding, grams	2952 ± 900
Age at discharge, days	61 (IQR 43 – 88)
Weight at discharge, grams	3391 ± 533

The included patients required a median of 41 days of parenteral nutrition (IQR 29 – 54), needing a central venous catheter (CVC) for parenteral nutrition and different treatments. The median duration of CVC use for all patients was 47.5 days (IQR 32.0–68.8). When stratified by etiology, patients with congenital chylothorax had a significantly longer median CVC duration of 83.5 days (IQR 52.3–140.0) compared to 39.5 days (IQR 32.0–65.0) in the acquired chylothorax group. However, the difference did not reach statistical significance (p = 0.099).

Fifteen patients developed late-onset sepsis, requiring a median of 18 days of antibiotics (IQR 9 – 31). All patients with acquired chylothorax were exposed to prophylactic antibiotics prior to diagnosis, whereas patients with congenital chylothorax received antibiotics only after the diagnosis was established.

Conversely, no patients received immunoglobulin or albumin treatment prior to our initial blood and chyle sampling.

### Comparison of peripheral blood and chyle immunological profile

**Figure 1:**
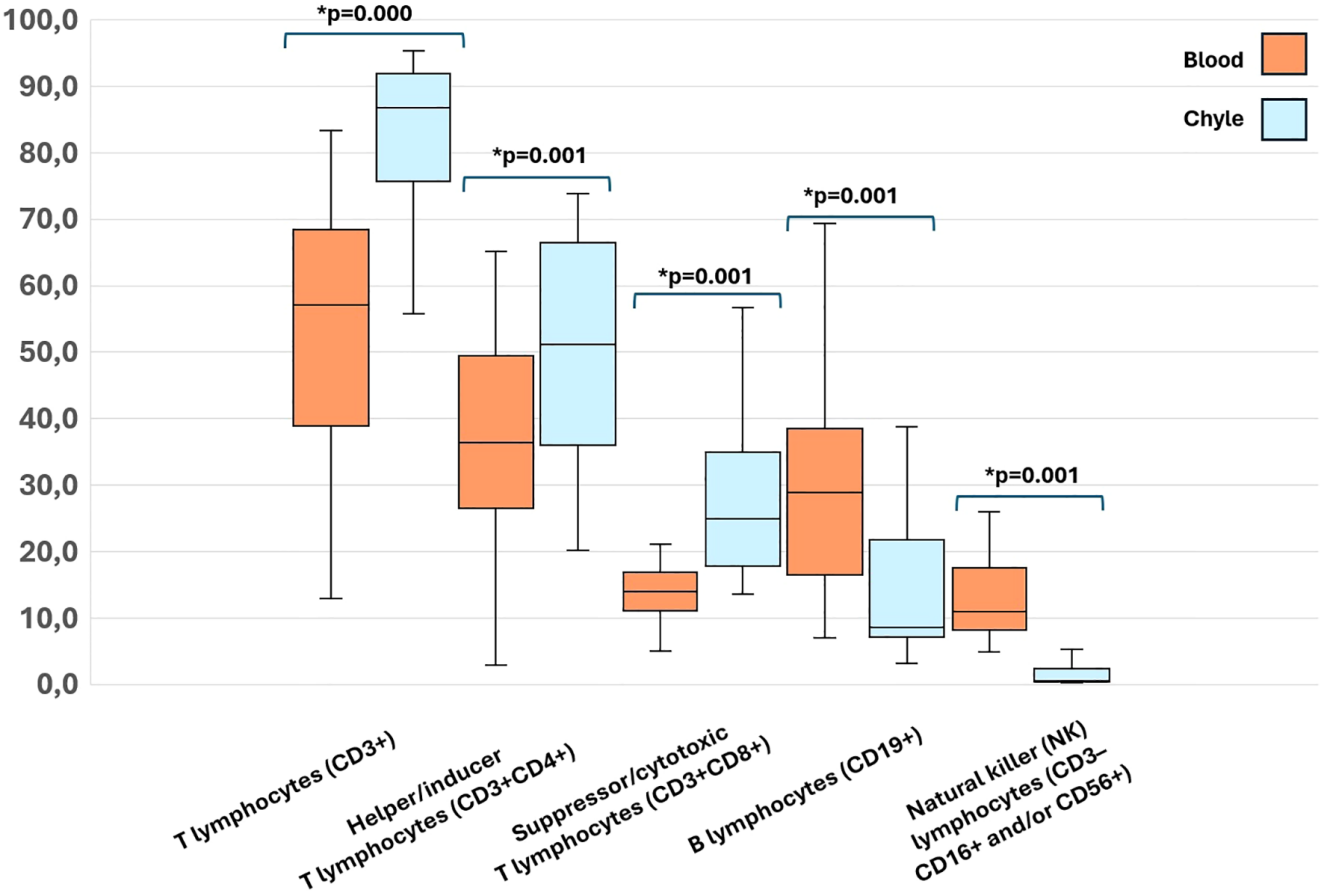
Significant differences in percentages of Lymphocyte subsets in paired peripheral blood and chyle samples of the included patients. A p-value < 0.05 was considered significant.

**Table 2** presents a comparison of lymphocyte subsets and immunoglobulins in paired peripheral blood and chyle samples from patients. We found a significantly higher leukocyte count, both the percentage and absolute count of lymphocytes, T lymphocytes (CD3^+^), Helper/inducer T lymphocytes (CD3^+^CD4^+^) and Suppressor/cytotoxic T lymphocytes (CD3^+^CD8^+^) in chyle. Interestingly, the chyle showed a significant enrichment of T lymphocytes compared to peripheral blood (**Figure 1**). Furthermore, IgG and IgM immunoglobulin levels were significantly lower in chyle.

**Table 2:** Lymphocyte subsets and immunoglobulins in paired peripheral blood and chyle samples of the included patients.

	Blood	Chyle	p-value
Leukocytes	990 (8580 – 14720)	4048 (2020 – 9706)	**0.010**
Lymphocytes (%)	13.8 (9.6 – 19.5)	81.0 (79.0 – 85.0)	**0.000**
Lymphocytes (cells/µl)	1630 (1290 – 2040)	3225 (1820 – 7864)	**0.010**
T lymphocytes (CD3^+^) (%)	57.0 (38.8 – 68.4)	86.7 (75.6 – 91.9)	**0.000**
T lymphocytes (CD3^+^) (cells/µl)	666 (431 – 1789)	1983 (1494 – 7440)	**0.001**
Helper/inducer T lymphocytes (CD3^+^CD4^+^) (%)	36.4 (26.4 – 49.4)	51.1 (35.9 – 66.4)	**0.001**
Helper/inducer T lymphocytes (CD3^+^CD4^+^) (cells/µl)	426 (288 – 1062)	1196 (765 – 4615)	**0.001**
Suppressor/cytotoxic T lymphocytes (CD3^+^CD8^+^) (%)	14.0 (11.1 – 16.8)	24.9 (17.7 – 34.9)	**0.001**
Suppressor/cytotoxic T lymphocytes (CD3^+^CD8^+^) (cells/µl)	222 (107 – 438)	865 (317 – 2184)	**0.001**
B lymphocytes (CD19^+^) (%)	28.8 (16.5 – 38.4)	8.5 (7.2 – 21.8)	**0.001**
B lymphocytes (CD19^+^) (cells/µl)	411 (176 – 531)	392 (167 – 953)	0.177
NK lymphocytes (CD3–CD16^+^ and/or CD56^+^) (%)	10.9 (8.1 – 17.5)	0.6 (0.4 – 2.4)	**0.001**
NK lymphocytes (CD3–CD16^+^ and/or CD56^+^) (cells/µl)	175 (132 – 286)	26 (16 – 51)	**0.004**
IgA (g/L)	0.04 (0.04 – 0.12)	0.04 (0.04 – 0.07)	0.127
IgG (g/L)	2.09 (0.91 – 3.47)	1.4 (0.30 – 2.29)	**0.004**
IgM (g/L)	0.23 (0.15 – 0.36)	0.08 (0.06 – 0.15)	**0.001**

Percentages for T, B, and NK cells represent the proportion of total lymphocytes, while percentages for CD4+ and CD8+ T cell subsets represent the proportion of CD3+ T cells. A p-value < 0.05 was considered significant.

Three patients (16.7%) presented with absolute lymphopenia. In **Table 3**, we reported how many patients had absolute and relative lymphopenia for the specific lymphocyte subsets, particularly within the T-cell and NK-cell subsets, whereas we found a complete absence of B-cell lymphopenia, revealing a specific pattern of immune dysregulation.

**Table 3:** Number of patients with absolute and relative lymphopenia for specific lymphocyte subsets in peripheral blood samples.

	Absolute lymphopenia	Relative lymphopenia
T lymphocytes (CD3^+^)	4 (22.2%)	2 (11.1%)
Helper/inducer T lymphocytes (CD3^+^CD4^+^)	7 (38.9%)	2 (11.1%)
Suppressor/cytotoxic T lymphocytes (CD3^+^CD8^+^)	6 (33.3%)	3 (16.7%)
B lymphocytes (CD19^+^)	0	0
NK lymphocytes (CD3–CD16^+^ and/or CD56^+^)	4 (22.2%)	1 (5.6%)

Furthermore, five patients (27.8%) had hypogammaglobulinemia. In total, eleven patients (61.1%) received intravenous immunoglobulins (IVIG) to maintain adequate blood IgG levels or IgM-enriched IVIG during late-onset sepsis episodes.

In **Table 4**, we compared lymphocyte subsets and immunoglobulins specifically within chyle samples from those with congenital chylothorax and those with acquired chylothorax. Data indicated that while the overall cellularity of chyle, particularly the absolute numbers of T-cell subsets (T lymphocytes (CD3^+^), Helper/inducer T lymphocytes (CD3^+^CD4^+^) and Suppressor/cytotoxic T lymphocytes (CD3^+^CD8^+^)), was significantly higher in acquired chylothorax, the relative proportions (percentages) of most lymphocyte subsets and the levels of immunoglobulins were largely similar between congenital and acquired chylothorax.

**Table 4:** Lymphocyte subsets and immunoglobulins in chyle samples from patients with congenital chylothorax and patients with acquired chylothorax.

	Chyle (congenital chylothorax) (n= 4)	Chyle (acquired chylothorax) (n= 14)	p-value
Leukocytes	1035 (851 – 2911)	6436 (2719 – 10750)	**0.035**
Lymphocytes (%)	79.5 (78.3 – 80.8)	82.0 (79.3 – 85.8)	0.382
Lymphocytes (cells/µl)	838 (692 – 2307)	4978 (2060 – 9062)	0.079
T lymphocytes (CD3^+^) (%)	75.9 (73.0 – 80.4)	90.5 (80.0 – 91.9)	0.382
T lymphocytes (CD3^+^) (cells/µl)	661 (464 – 1852)	3860 (1640 – 8329)	**0.035**
Helper/inducer T lymphocytes (CD3^+^CD4^+^) (%)	37.8 (25.9 – 54.3)	56.6 (38.4 – 66.4)	0.442
Helper/inducer T lymphocytes (CD3^+^CD4^+^) (cells/µl)	427 (189 – 1269)	2183 (1079 – 5064)	**0.046**
Suppressor/cytotoxic T lymphocytes (CD3^+^CD8^+^) (%)	30.7 (23.5 – 37.8)	23.6 (17.8 – 32.6)	0.574
Suppressor/cytotoxic T lymphocytes (CD3^+^CD8^+^) (cells/µl)	212 (127 – 661)	1023 (506 – 2281)	**0.046**
B lymphocytes (CD19^+^) (%)	20.5 (15.5 – 24.6)	8.3 (7.2 – 18.4)	0.382
B lymphocytes (CD19^+^) (cells/µl)	156 (52 – 571)	474 (285 – 953)	0.277
NK lymphocytes (CD3–CD16^+^ and/or CD56^+^) (%)	1.3 (0.6 – 2.7)	0.5 (0.3 – 2.3)	0.442
NK lymphocytes (CD3–CD16^+^ and/or CD56^+^) (cells/µl)	15 (11 – 19)	40 (19 – 73)	0.079
IgA (g/L)	0.04 (0.04 – 0.06)	0.04 (0.04 – 0.07)	0.878
IgG (g/L)	0.30 (0.25 – 0.59)	1.56 (0.44 – 2.43)	0.127
IgM (g/L)	0.07 (0.05 – 0.08)	0.11 (0.07 – 0.17)	0.233

A p-value < 0.05 was considered significant.

As shown in **Table 5**, neonates with acquired chylothorax seemed to have higher absolute numbers of lymphocytes and T-cell subsets (CD3^+^, CD3^+^CD4^+^, CD3^+^CD8^+^) compared to those with congenital chylothorax, although not significantly.

**Table 5:** Lymphocyte subsets and immunoglobulins in blood samples from patients with congenital chylothorax and patients with acquired chylothorax.

	Chyle (congenital chylothorax) (n= 4)	Chyle (acquired chylothorax) (n= 14)	p-value
Leukocytes	1290 (1025–1460)	1795 (1528–3045)	0.165
Lymphocytes (%)	13.8 (10.6–15.9)	13.7 (9.7–22.7)	0.676
Lymphocytes (cells/µl)	1290 (1025–1460)	1795 (1528–3045)	0.165
T lymphocytes (CD3^+^) (%)	35.2 (24.1–37.3)	65.1 (55.1–72.3)	0.047
T lymphocytes (CD3^+^) (cells/µl)	387 (245–512)	878 (507–1853)	0.121
Helper/inducer T lymphocytes (CD3^+^CD4^+^) (%)	26.4 (14.7–26.9)	40.7 (33.5–51.0)	0.068
Helper/inducer T lymphocytes (CD3^+^CD4^+^) (cells/µl)	302 (163–364)	643 (301–1089)	0.197
Suppressor/cytotoxic T lymphocytes (CD3^+^CD8^+^) (%)	6.3 (5.7–8.3)	14.3 (12.8–20.0)	0.006
Suppressor/cytotoxic T lymphocytes (CD3^+^CD8^+^) (cells/µl)	55 (53–111)	254 (162–439)	0.091
B lymphocytes (CD19^+^) (%)	38.4 (37.9–44.9)	20.8 (16.1–33.3)	0.121
B lymphocytes (CD19^+^) (cells/µl)	411 (411–516)	352 (169–524)	0.571
NK lymphocytes (CD3–CD16^+^ and/or CD56^+^) (%)	26.0 (23.6–30.6)	10.8 (7.7–15.1)	0.014
NK lymphocytes (CD3–CD16^+^ and/or CD56^+^) (cells/µl)	286 (284–314)	153 (100–242)	0.121
IgA (g/L)	0.04 (0.04–0.08)	0.04 (0.04–0.13)	0.724
IgG (g/L)	0.66 (0.48–0.92)	2.89 (1.65–3.72)	0.051
IgM (g/L)	0.15 (0.10–0.15)	0.29 (0.20–0.38)	0.038

### Late-onset sepsis and hypogammaglobulinemia

Fifteen infants (83.3%) had at least one late-onset sepsis episode during NICU stay: 11/15 patients (73.3%) had one episode, 2/15 patients (13.3%) had three episodes, one patient (6.7%) had four episodes, and one patient (6.7%) had seven episodes. Involved pathogens were both Gram-positive bacteria (coagulase-negative Staphylococci and *Staphylococcus aureus*) and Gram-negative bacteria (*Klebsiella pneumoniae*, *Enterobacter* spp., *Serratia marcescens*, *Stenotrophomonas maltophilia*, *Proteus mirabilis*); fungi were identified only in two cases (*Candida parapsilosis* and *Candida lusitaniae*) (11.1%). A further patient (5.5%) had no late-onset sepsis episodes but had one pneumonia episode caused by *Staphylococcus haemolyticus*.

Finally, we found that infants with hypogammaglobulinemia had a higher mean of late-onset sepsis episodes (2.00 ± 2.83) than those without (1.32 ± 1.19), although not significantly (p=0.070). Similarly, infants who received IVIG had a greater mean of late-onset sepsis episodes (1.82 ± 2.1) than those who did not (1.14 ± 0.9), although not significantly (p=0.076).

## Discussion

This study, investigating lymphocyte subsets and immunoglobulin levels in neonates and infants with chylothorax, revealed several key findings directly addressing our primary aim. We observed a significant differential distribution of immune cells, with chyle showing a higher concentration of leukocytes, total lymphocytes, T lymphocytes (CD3^+^), Helper/inducer T lymphocytes (CD3^+^CD4^+^) and Suppressor/cytotoxic T lymphocytes (CD3^+^CD8^+^) compared to peripheral blood. Conversely, B lymphocytes and NK cells were notably depleted in chyle. Furthermore, IgG and IgM levels were significantly lower in chyle, indicating a potential local immune compromise or preferential trafficking of specific immunoglobulin classes. Interpreting Ig levels in the first months of life demands caution due to their pronounced age-dependent variability. Newborns’ serum IgG levels are comparable to their mothers’ at birth because nearly all IgG is maternally acquired. However, maternal IgG declines postnatally, reaching a low point at 6–12 months (15).

Chylothorax in neonates and infants is not merely a mechanical issue of fluid accumulation but also alters systemic and local immunity (16).

Drainage of chylothorax may be required for symptomatic relief, though it carries some risks. Prolonged chest tube drainage may result in immunoglobulin and lymphocyte loss, contributing to immunosuppression (17). In particular, the distinct immunological profile that we found in chyle, particularly the depletion of B lymphocytes and NK cells and lower immunoglobulin levels, supports the hypothesis that the lymphatic fluid environment might contribute to increased susceptibility to infections, especially in the case of impaired lymphatic drainage (8).

Previously, Hoskote et al. evaluated whether intravenous immunoglobulin administration was linked to a reduction in sepsis in children with prolonged chylothorax after cardiothoracic surgery. They reported that patients with prolonged, large-volume chyle loss had greater “secondary immunodeficiency”, supporting the idea of lower immunoglobulin levels in chyle, particularly with significant loss. The sample size was small, and the authors were not able to detect a large treatment effect from intravenous immunoglobulin, because infectious outcomes were equal between those who received supplementation and those who did not (9).

Conversely, Orange et al. evaluated humoral and cellular immunity in 8 patients with chylothorax, also analyzing chylous fluid to document cellular losses (8). While there were differences in patient demographics and specific findings in our cohort and in the one reported by Orange et al., we provided data from a larger cohort and a more detailed breakdown of lymphocyte subsets in both peripheral blood and chyle. Both studies confirmed that chylothorax was associated with significant alterations in immune status, with depletion of immunoglobulins (IgG and IgM in our study, IgG in the cohort by Orange et al.) and significant differences in lymphocyte populations between peripheral blood and chyle, with lymphocytes T more prevalent in chyle (8). Orange et al. specifically highlighted that the ratio of CD3+/CD45RA+ naive: CD3+/CD45RO+ memory T cells was greater in chyle than in peripheral blood, suggesting a preferential loss of naive T cells from the circulation into the chyle (8). Our study did not assess naive/memory subsets.

While all 8 patients in Orange et al.’s cohort had both hypogammaglobulinemia and lymphopenia, these were observed in 27.8% and 16.7% of our patients, respectively (8).

The high incidence of late-onset sepsis in these two cohorts (83.3% in our cohort and 75% in the cohort by Orange et al.) further underscores the critical need for heightened vigilance and potentially targeted immune support strategies in this vulnerable patient population.

Waterhouse et al. found a greater rate of central line-associated bloodstream infections in young patients with chylothorax following cardiac surgery. In these patients with chylothorax, the complexity of surgery, central venous catheter duration, and chest tube output were associated with increased risk for developing a central line–associated bloodstream infection (18). Indeed, the incidence of any infection can be high in infants undergoing major surgery, with a consequent high mortality (12, 19–21), with a higher rate in those with acquired chylothorax, which could be due to decreased immunological and cellular immunity.

Wasmuth‐Pietzuch et al. also reported a high incidence of nosocomial infections (57%) in 7 infants with congenital chylothorax, with rates approximately three times higher than those in other neonatal patients (5).

In particular, we specified that not all lymphocyte populations were equally impacted. The complete absence of B-cell lymphopenia contrasted with the high frequency of T-cell lymphopenia, revealing a specific pattern of immune dysregulation. These findings enhance understanding of immune alterations in chylothorax and may inform future infection prevention strategies.

Addressing our secondary aim, while the absolute numbers of T-cell subsets were higher in acquired chylothorax, the relative proportions of most lymphocyte subsets and immunoglobulin levels within chyle were largely similar between congenital and acquired forms. This can be due to the composition of chyle, irrespective of its origin (congenital or acquired), which is determined by its physiological role (transport of lipids, fat-soluble vitamins, lymphocytes, and immunoglobulins from the gastrointestinal tract to the bloodstream) (17). The cause of the leak (congenital malformation vs. traumatic injury) seemed not to alter the global nature of the fluid itself, but rather the fluid amounts in the pleural space. The higher cellularity in acquired chylothorax could be explained by a more acute inflammatory response following a surgical event, which would lead to an abrupt accumulation of immune cells to the site of injury. In contrast, congenital chylothorax, often resulting from a malformation, may involve a more chronic, steady-state leakage of lymphatic fluid, which could account for the lower absolute lymphocyte counts.

However, since the composition of the fluid itself (in terms of relative proportions and immunoglobulin levels) remains similar, our findings suggest that the underlying immune mechanism of chyle leakage, regardless of its origin, has a consistent effect on the immune landscape.

It’s also interesting to analyze the pathogens’ spectrum, with late-onset sepsis episodes mainly due to bacteria, and some fungal cases; no fungal infections were previously reported (5).

The limitations of our study are linked to the observational retrospective design and the small sample size, though this is a relatively adequate number for a rare condition like chylothorax in neonates. This limits the generalizability of our findings, particularly in subgroup analyses between congenital and acquired chylothorax. Furthermore, our study included all consecutive patients with chylothorax who underwent pleural drainage during the study period. Patients who were managed conservatively (i.e., without drainage) were not included in this analysis, as our data collection was specifically focused on samples obtained from pleural drainage. Unfortunately, we do not have a general registry of all acquired chylothorax cases treated at our institution, which makes it impossible for us to provide the exact denominator of all chylothorax patients, including those managed conservatively. Furthermore, the laboratory assays we used are validated for blood analyses, and we were able to apply them successfully to chyle. However, since chylothorax is a rare condition, we did not have access to “healthy” pleural fluid controls for direct comparison. Additionally, cell concentrations in chyle provided only a partial view of lymphocyte and immunoglobulin loss. A more informative measure would be the total number lost over time (concentration × chyle volume). In this retrospective study, daily chyle volumes were not consistently recorded, preventing such calculations. Future prospective studies should include systematic chyle volume measurements to better estimate total immune cell and immunoglobulin loss and its clinical impact. Moreover, a more detailed characterization of lymphocyte subsets would have been of interest, but due to the limited sample availability this was not feasible.

Conversely, we found that hypogammaglobulinemia was associated with a greater mean of late-onset sepsis episodes, although not significantly. This does not automatically imply causality but rather suggests that hypogammaglobulinemia could be an effective indicator of the risk of sepsis, even more so given that we performed immunoglobulin measurements in paired peripheral blood and chyle samples on the same day, before IVIG administration.

## Conclusion

Our findings confirm the presence of specific lymphocyte subsets and immunoglobulins in the pleural fluid in patients with chylothorax, with a specific pattern of immune dysregulation, especially within the T-cell subsets that were enriched in the chyle. Nevertheless, the clinical significance of these quantitative alterations remains to be further investigated, and the opportunity to supplement immunoglobulins in these infants should be studied in a multicenter, prospective, randomized controlled trial, to determine whether immunoglobulin supplementation may be beneficial before the onset of hypogammaglobulinemia.

## Data Availability

The original contributions presented in the study are included in the article/supplementary material. Further inquiries can be directed to the corresponding author.
